# Cell Type‐Specific Extracellular Vesicles in Mouse Brain: Proteomic Signatures Highlight Astrocytic GlialCAM Network and GPCR Enrichment

**DOI:** 10.1002/glia.70212

**Published:** 2026-08-21

**Authors:** Alba M. Lucart‐Sanchez, Edward Sellés‐Climent, Jorge Navarro‐Calvo, Guillem Pont‐Espinós, Jose A. Gomez‐Sanchez, Raúl Estévez, Luis M. Valor, Rocío Pérez‐González

**Affiliations:** ^1^ Instituto de Investigación Sanitaria y Biomédica de Alicante (ISABIAL) Alicante Spain; ^2^ Instituto de Neurociencias de Alicante, Universidad Miguel Hernández‐CSIC San Juan de Alicante Spain; ^3^ Centro de Investigación en Red‐Enfermedades Neurodegenerativas (CIBERNED) Madrid Spain; ^4^ Physiology Unit, Department of Physiological Sciences School of Medicine and Health Sciences, Institute of Neurosciences, University of Barcelona L'Hospitalet de Llobregat Spain; ^5^ Neuroscience Program, Physiology and Pathology of the Functional Relationship Between Glia and Neurons‐IDIBELL L'Hospitalet de Llobregat Spain; ^6^ The Spanish Center of Rare Diseases (CIBERER U‐731) Barcelona Spain; ^7^ Instituto de Investigación, Desarrollo e Innovación en Biotecnología Sanitaria de Elche (IDiBE) Elche Spain

**Keywords:** astrocytes, blood–brain barrier, extracellular vesicles, GlialCAM, GPCR, proteomics

## Abstract

Extracellular vesicles (EVs) mediate intercellular communication in the central nervous system (CNS) and are emerging as biomarkers of brain health and disease. However, the molecular composition of cell type‐specific brain EVs, particularly astrocyte‐derived EVs (ADEVs), remains poorly defined. We performed comparative proteomic analysis of neuronal (NDEVs), microglial (MDEVs), and ADEVs from mouse brain using magnetic immunocapture and LC–MS/MS proteomic profiling. Each EV subtype displayed distinct molecular fingerprints. NDEVs were enriched in synaptic and neurogenesis‐related proteins (e.g., APP, SNAP25, GPR158, and BDNF), whereas MDEVs contained immune and phagocytic markers (e.g., TMEM119, CX3CR1, CD11b). Strikingly, the ADEV proteome closely mirrored the recently characterized GlialCAM interactome from leukodystrophy research, encompassing GlialCAM/MLC1 and associated partners involved in ion and water homeostasis (EAAT1/2, AQP4, GJA1), together with GPCRs such as GPRC5B. This overlap suggests that ADEVs encapsulate a molecular scaffold characteristic of astrocytic endfeet, potentially extending their signaling functions to the extracellular space. In conclusion, our study provides a detailed comparative proteomic characterization of brain cell type‐specific EVs, revealing that ADEVs contain the GlialCAM/MLC1 network and GPCRs. These findings identify candidate molecular signatures that support the future investigation of ADEVs for biomarker development and provide a proteomic framework for exploring their relationship with astrocytic endfoot biology, blood–brain barrier (BBB)‐associated pathways, and neurodegenerative disorders.

## Graphical abstract

- Cell type–specific EVs were isolated from mouse brain and profiled by proteomics.
- Each EV subtype exhibited proteomic signatures consistent with the specialized functions of its cell of origin.
- Astrocyte‐derived EVs (ADEVs) were enriched for the GlialCAM/MLC1 network and GPCRs.
- ADEV cargo captured molecular features of astrocytic endfoot functions and BBB‐related pathways.

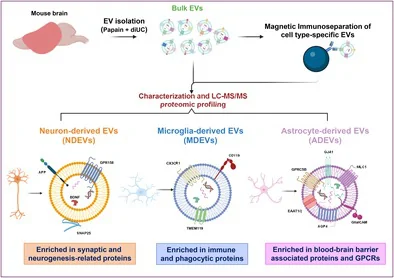

## 1 Introduction

Extracellular vesicles (EVs) are nanosized, lipid‐bilayer particles secreted by all cell types in the central nervous system (CNS), including neurons, astrocytes, and microglia. They carry proteins, lipids, and nucleic acids reflective of their cells of origin, thereby serving as dynamic mediators of intercellular communication and signaling (Yáñez‐Mó et al. 2015). Within the brain, EVs play essential roles in maintaining CNS homeostasis and facilitating neurovascular and neuroimmune signaling (Ikezu et al. 2024). Emerging evidence suggests that certain EV subpopulations can cross the blood–brain barrier (BBB), enabling molecular exchange between the CNS and peripheral circulation under both physiological and pathological conditions (Alvarez‐Erviti et al. 2011; Filannino et al. 2024; Valadi et al. 2007; Wang et al. 2023). This property positions brain EVs as promising, minimally invasive biomarkers of CNS diseases, neuroinflammation, and BBB function. Despite this potential, isolating cell type–specific brain EVs from biofluids remains a major technical challenge. This is because EV surface proteins that enable antibody‐based immunocapture must be both cell‐specific and extracellularly accessible, criteria that are difficult to satisfy given the molecular overlap between CNS and peripheral EVs (Newman and Rowland 2025). Moreover, the intrinsic cellular heterogeneity of the brain, encompassing diverse neuronal and glial populations, further complicates efforts to achieve precise and reproducible identification of EV subtypes.

To date, neuron‐derived EVs (NDEVs) have primarily been isolated using L1CAM; however, the predominance of soluble L1CAM fragments over full‐length forms has raised concerns regarding its specificity (Kadam et al. 2024; Norman et al. 2021). Additional neuronal markers, such as ATP1A3, have shown promise for improving NDEVs isolation (You et al. 2023). For microglia‐derived EVs (MDEVs), CD11b (Cohn et al. 2021; Melnik et al. 2024) and TMEM119 (Kumar et al. 2021; Roseborough et al. 2023) have been used. In contrast, relatively few studies have systematically examined astrocyte‐derived EVs (ADEVs), despite astrocytes' central roles in neurovascular coupling, BBB integrity, and metabolic support of neurons. Markers such as EAAT1, EAAT2, and ATP1B2 have been used to enrich for ADEVs (Valle‐Tamayo et al. 2022; You et al. 2022; Zhang, Lobb, et al. 2025), with EAAT1 emerging as the most widely applied (Badhwar et al. 2024), while Aquaporin‐4 (AQP4) has been proposed as a new immunocapture target (You et al. 2022). However, the surface proteome of ADEVs isolated from brain tissue, particularly proteins associated with astrocytic endfeet and neurovascular interfaces, remains incompletely defined.

Here, we address this knowledge gap by performing a comparative proteomic analysis of three major brain EV subtypes: neuronal (NDEVs), microglial (MDEVs), and astrocytic (ADEVs), isolated from the same mouse brains using immunocapture and LC–MS/MS. Our results reveal distinct molecular signatures that reflect the specialized physiological roles of each parent cell type. Notably, ADEVs exhibit a proteomic profile that strongly overlaps with the GlialCAM interactome, including GlialCAM/MLC1, GPCRs, and transporters/ion channels, suggesting that ADEVs selectively encapsulate components of astrocytic endfeet function and signaling hubs. These findings provide a proteomic framework for refining immunocapture strategies and advancing the investigation of ADEVs as candidate biomarkers of CNS pathology and a window into astrocytic endfoot biology and BBB‐associated pathways.

## 2 Materials and Methods

### 2.1 Mouse Housing and Brain Tissue Collection

C57BL/6J young adults were housed under a 12‐h light/dark cycle with food and water provided ad libitum at the animal facilities of Servicios Técnicos de Investigación, Universidad de Alicante. After sacrifice, mouse brains were immediately dissected and snap‐frozen. All procedures were approved by the Comité de Ética de la Investigación, Universidad de Alicante, and authorized by the Dirección General de Producción Agrícola y Ganadera, Generalitat Valenciana, according to European, national, and regional laws.

### 2.2 EV Isolation

Frozen mouse brains, without the cerebellum and olfactory bulbs, from female and male young adults, were used for EV isolation and characterization. Small EVs were isolated as previously described (Pérez‐González et al. 2012; Pérez‐González et al. 2017) with some modifications. Briefly, the tissues (~300 mg) were minced and incubated with 20 U/mL papain (Worthington Biochemical Corporation, LK003176) and 40 U/mL DNAse I (Roche, 11284932001) in Hibernate A medium (Thermo Fisher Scientific, A12475‐01) for 15 min at 37°C. The enzymatic digestion was stopped by the addition of ice‐cold Hibernate A supplemented with a cocktail of protease and inhibitors (5 μg/mL leupeptin, 5 μg/mL antipain dihydrochloride, 5 μg/mL pepstatin A, 1 mM phenylmethylsulfonyl fluoride (PMSF), and 1 μM E‐64, all from Sigma‐Aldrich). The solution was gently dissociated by pipetting and centrifuged at 300 × *g* for 10 min at 4°C to pellet undigested tissue and intact cells. The supernatant was subsequently filtered through a 40 μm cell strainer and then centrifuged at 2000 × *g* for 10 min at 4°C to discard large debris and apoptotic bodies. The resulting supernatant was centrifuged at 10,000 × *g* for 30 min at 4°C to discard smaller debris, passed through a 0.2 μm surfactant‐free cellulose acetate (SFCA) membrane filter (Corning, 431219), and ultra‐centrifuged at 100,000 × *g* for 70 min at 4°C to pellet EVs in a Beckman Coulter 70 Ti fixed‐angle rotor (k factor 106). The pellet was washed in phosphate‐buffered saline (PBS), re‐centrifuged at 100,000 × *g* for 70 min at 4°C and resuspended in PBS by gentle pipetting to obtain a homogeneous EV suspension for subsequent immunocapture steps.

### 2.3 Immunocapture of Cell Type–Specific EVs (ATP1A3 ^+^, CD11b ^+^, ACSA‐2^+^)

Magnetic beads (Invitrogen, 14311D) were conjugated with anti‐ATP1A3 (Invitrogen, MA3‐915) and anti‐CD11b (BioLegend, 101201) for NDEVs and MDEVs, respectively, at a ratio of 5 μg of antibody per mg of beads. ADEVs were immunocaptured using the anti‐ACSA‐2 Microbead Kit (Miltenyi Biotec, 130‐097‐679). Brain EVs (45 μg per immunocapture, corresponding to the EV yield obtained from approximately 90 mg of tissue) were incubated with 10 μL of FcR Blocking Reagent, Mouse (Miltenyi Biotec, 130‐092‐575) for 15 min on ice. Subsequently, 100 μL of antibody‐conjugated magnetic beads were added for overnight incubation at 4°C under rotation in a final volume of 500 μL. To assess non‐specific binding, magnetic beads conjugated with an IgG control antibody (R&D Systems, MAB002) were incubated with brain EVs under identical conditions. Bulk EVs and unbound fractions corresponding to the non‐immunocaptured EVs were kept for assessing immunocapture efficiency, while beads incubated with only PBS (1×) were used as negative controls. For electron microscopy, nanoparticle tracking analysis (NTA), and mass spectrometry, cell type–specific EVs were released from magnetic beads using IgG Elution Buffer (Thermo Fisher Scientific, 21028), followed by pH neutralization with 1 M Tris base buffer.

### 2.4 Sample Lysis, Protein Estimation and Western Blotting

Tissue pellets and EVs were lysed in 2× RIPA buffer (except for immunocaptured EVs that were eluted in 1× RIPA) supplemented with protease inhibitors and sonicated for 45 s in a water bath at 4°C followed by incubation on ice for 20 min with brief vortexing every 2–3 min. The first pellets obtained after centrifugation at 300 × *g* were mechanically dissociated using a pestle on ice before sonication, centrifuged at 15,700 × *g* for 15 min at 4°C, and the supernatant lysed for protein analysis. Protein concentrations were determined using the Pierce BCA Protein Estimation Assay Kit (Thermo Fisher Scientific, 23227). Lysates were then denatured in 6X Laemmli SDS sample buffer (Thermo Fisher Scientific, J61337‐AC), under reducing conditions, and proteins were separated by SDS–PAGE using 10% TGX Stain‐Free Fast Cast acrylamide gels (Bio‐Rad, catalog no. 1610183). Magnetic beads (except microbeads) were removed before SDS‐PAGE. Proteins were transferred onto 0.45 μm PVDF membranes using the Trans‐Blot Turbo Mini‐Size LF PVDF Transfer Pack (Bio‐Rad, 10026934). Membranes were blocked with EveryBlot Blocking Buffer (Bio‐Rad, 12010020) and incubated overnight at 4°C with the following primary antibodies: anti‐ATP1A3 (Rabbit, 1:1000, Proteintech, 10868‐1‐AP), anti‐Glutamine Synthetase (Rabbit, 1:1000, GeneTex, GTX109121), anti‐Alix (Rabbit, 1:1000, Cell Signaling, 92880S), anti‐CD11b (Rabbit, 1:1000, GeneTex, GTX640543), anti‐Calnexin (Rabbit, 1:20.000, GeneTex, GTX109669), anti‐Myelin Basic Protein (MBP) (Mouse, 1:700, Biolegend, 836501), anti‐Lamin A/C (Mouse, 1:1000, Cell Signaling, 4777), anti‐Synaptophysin (Rabbit, Thermo Fisher Scientific, MA5‐14532), anti‐COX IV (Rabbit, GeneTex, GTX114330), and anti‐VDAC (Rabbit, Cell Signaling, 4866). For the analysis of the astrocytic GlialCAM interactome proteins, we used anti‐GlialCAM (López‐Hernández et al. 2011), anti‐GPRC5B (Alonso‐Gardón et al. 2021) and anti‐MLC1 (Teijido et al. 2004), all Rabbit at 1:500 dilution. After washing, membranes were incubated for 1 h at room temperature with horseradish peroxidase (HRP)‐conjugated secondary antibody (Cell Signaling, 7074S or 7076S). Protein bands were visualized using SuperSignal West Femto Maximum Sensitivity Substrate (Thermo Fisher Scientific, A38554) and imaged with the ChemiDoc MP Imaging System (Bio‐Rad). Gels (after electrophoresis) or membranes (before blocking) were also imaged for total protein measurement. Densitometric analyses were performed using ImageJ software (NIH). Uncropped blots are shown in Supporting Information.

### 2.5 Scanning Transmission Electron Microscopy (STEM) of EV Preparations

EV imaging was performed at the Microscopy Unit of ISABIAL. For STEM sample preparation, 10 μL of EV suspension was placed onto Formvar/Carbon‐coated copper grids (200 mesh) and allowed to adsorb for 25 min. Excess liquid was gently removed with filter paper. To enhance surface contrast, the samples were negatively stained with 10 μL of 2% uranyl acetate (pH 7.4) for 20 s. The excess stain was carefully removed without directly contacting the grid surface. After complete air‐drying, the grids were mounted in a dedicated STEM grid holder and examined using a field emission scanning electron microscope (FE‐SEM, Zeiss GeminiSEM 460) equipped with a STEM detector operating at 30.0 kV. Images were acquired at magnifications of 10 and 3 μm to assess EV morphology and surface characteristics.

### 2.6 NTA

The particle size distribution and concentration measurements were evaluated with a Nanosight NS300 (Particle Tracking Analysis) instrument (Malvern Panalytical, Malvern, UK). EVs were resuspended in PBS and diluted to the working range of the system (106–109 particles/mL). Videos were captured and analyzed with the Nanosight NS300 software (version 3.4) using a sCMOS camera.

### 2.7 Mass Spectrometry and Proteomic Data Analysis

Mass spectrometry and proteomic analyses were performed at the proteomics facility of SCSIE University of Valencia, a member of Proteored. All protein extracts were concentrated to 20 μL using a SpeedVac and subsequently mixed with 25 μL of 4× Laemmli sample buffer (Bio‐Rad, 1610747). Samples were vortexed for 5 min and heated at 95°C for 5 min to ensure complete denaturation. The resulting protein extracts were loaded onto SDS–PAGE gels to assess sample quality, visualize protein bands, and estimate relative protein concentration.

Tandem mass spectrometry (LC–MS/MS) analysis was performed using a NanoElute2 HPLC system (Bruker) coupled to a timsTOF SCP mass spectrometer (Bruker). A total of 50 ng of digested peptides were separated on an Aurora Ultimate C18 analytical column (25 cm × 75 μm, 1.7 μm; Bruker Daltonics) using a 66‐min linear gradient on the NanoElute2 system. Peptides were ionized via CaptiveSpray at 1700 V and 200°C and analyzed in data‐dependent acquisition mode (ddaPASEF) using the method SCP_dda_low_SampleAmount_5PASEF. Instrument parameters included a 1/K₀ range of 0.7–1.3 V·s/cm^2^, ramp time of 166 ms, duty cycle of 100%, ramp rate of 5.78 Hz, MS averaging of 1, and auto‐calibration disabled. The MS scan range was set to 100–1700 m/z with positive ion polarity and PASEF scan mode. MS/MS acquisition included five PASEF ramps per cycle with a total cycle time of 1.04 s, charge range of 0–5, target intensity of 20,000, an intensity threshold of 500, and active exclusion enabled.

Raw data files were analyzed using FragPipe to identify proteins and perform label‐free quantification (LFQ) using standard in‐house parameters. The combined_protein.tsv output file was used for statistical analysis. Common contaminants and species‐mismatched proteins were excluded from the analysis. Proteins detected by MaxLFQ intensity in at least one of the three biological replicates were retained. MaxLFQ intensity values were log_2_‐transformed, and missing values were imputed using the Missing Not At Random (MNAR) approach, which samples random values from a left‐shifted Gaussian distribution (1.8 standard deviations downshift, width = 0.3). Differential expression analysis was performed using protein‐wise linear models combined with empirical Bayes statistics implemented in the *limma* package (R/Bioconductor). Proteins with an adjusted *p* value < 0.05 (FDR correction) and an absolute log_2_ fold change ≥ 1 were considered significantly differentially enriched. Venn diagrams were generated using InteractiVenn (Heberle et al. 2015), gene ontology (GO) enrichment analysis was performed with ShinyGO 0.82 (Ge et al. 2020), and protein–protein interaction (PPI) networks were constructed using STRING v12.0 (Szklarczyk et al. 2023). The heatmaps were generated using GraphPad Prism (version 8.01; GraphPad Software, San Diego, CA, USA) except for Figure 3G,H, which was made with Excel. The volcano plots were generated using SRplot (Tang et al. 2023).

The mass spectrometry proteomics data have been deposited to the ProteomeXchange Consortium via the PRIDE (Perez‐Riverol et al. 2025) partner repository with the dataset identifier PXD071055.

### 2.8 Meta‐Analysis With Gene Expression Datasets

Differentially enriched proteins were screened for cell‐specific markers that derived from a curated list of 100‐gene markers for neurons, astrocytes, oligodendrocytes, endothelial cells and microglia that are shared between humans and mice and that have been further validated in the literature (supplemental file 2 of the original publication (McKenzie et al. 2018)). To infer the cellular origin of the EV‐derived proteins, we used the median gene expression from single‐cell transcriptomics of mouse brains (cortex and hippocampus) for the Allen Brain Atlas (https://brain‐map.org/our‐research/cell‐types‐taxonomies/cell‐types‐database‐rna‐seq‐data/mouse‐whole‐cortex‐and‐hippocampus‐10x), according to the last cellular taxonomy (Yao et al. 2021). Genes with median expression = 0 were removed, assuming an issue regarding sequencing coverage.

### 2.9 Statistical Analysis

All statistical analyses were conducted using GraphPad Prism software (version 8.01; GraphPad Software, San Diego, CA, USA). A *p* value < 0.05 was considered statistically significant.

## 3 Results

### 3.1 Cell Type‐Specific EVs Immunocapture Validation

To characterize cell type‐specific small EVs from brain tissue, we optimized a workflow to enrich in neuronal, microglial, and astrocytic EVs, hereafter referred to as NDEVs, MDEVs, and ADEVs, respectively, following Minimal information for studies of EVs (MISEV2023) guidelines (Welsh et al. 2024) and recommendations for EV studies from solid tissue (Crescitelli et al. 2025). EV isolation was performed by differential ultracentrifugation followed by immunocapture of specific EVs (Figure S1). Throughout the isolation process, intermediate pellets were kept and analyzed by Western blot to assess the purity of the 100,000 × *g* pellet corresponding to bulk EVs. Bulk EVs were enriched in the canonical EV markers Alix and CD63 (Figure S2A,B). Calnexin, an intracellular marker, was mostly excluded from bulk EVs, confirming minimal intracellular contamination (Figure S2A,B). In addition, the nuclear marker lamin A/C, the mitochondrial markers COX IV and VDAC, the myelin marker MBP, and the synaptic marker synaptophysin were also depleted from EVs (Figure S2C,D).

**FIGURE 1:**
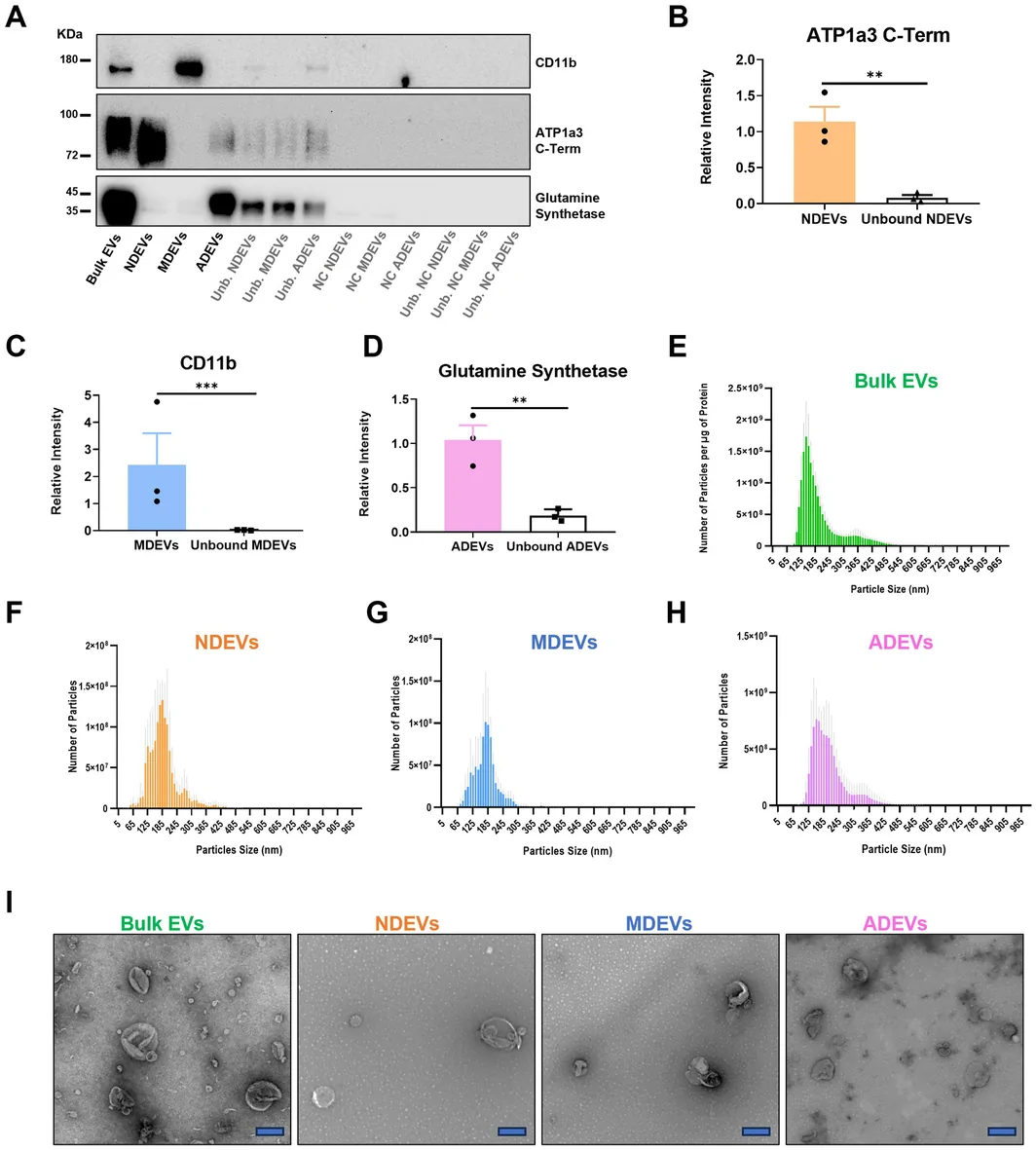
Cell type‐specific EVs immunocapture validation. (A) Representative Western blot showing enrichment of cell‐specific markers: CD11b in MDEVs (CD11b+EVs), ATP1A3 in NDEVs (ATP1A3+EVs) and glutamine synthetase in ADEVs (ACSA‐2+EVs), compared to bulk EVs. Unbound (Unb.) fractions are also shown. Beads conjugated with the respective antibodies and incubated with only PBS (1×) were used as negative controls (NC). (B‐D) Quantification of the relative intensity (mean ± SD) of immunocaptured EVs (bound) versus unbound fractions confirmed the efficiency of the immunocapture. Student *t*‐test, *N* = 3, **p < 0.01, ***p < 0.001. (E‐H) Size and number distributions among bulk and cell type–specific EV populations by nanoparticle tracking analysis (NTA). Data are presented as the mean number of particles at each bin center (colored bars) ± SEM (standard error of the mean; light gray bars), *N* = 4. The number of particles in bulk EVs was normalized by total μg of EV protein estimated by brain weight. (I) Scanning transmission electron microscopy (STEM) images show vesicles with size and morphology consistent with EVs in bulk and cell type‐specific preparations. Scale bars = 200 nm.

Western blot analysis verified the enrichment of ATP1A3, CD11b and glutamine synthetase in NDEVs, MDEVs and ADEVs, respectively (Figure 1A–D). NTA revealed distinct size and number distributions among EV populations (Figure 1E–H). All EV subtypes exhibited a predominant size below 200 nm, with MDEVs exhibiting a slightly larger modal size at 181 ± 20.12 nm (Figure 1G). In terms of EV numbers, ADEVs displayed the highest particle concentration of 8.87 × 10^9^ particles/mL, followed by NDEVs (1.32 × 10^9^ particles/mL) and MDEVs (8.43 × 10^8^ particles/mL). Lastly, electron microscopy (EM) imaging corroborated the presence of intact vesicles with morphology and size consistent with small EVs in all populations (Figure 1I). Wider field EM images confirmed the presence of EVs distributed throughout all the preparations (Figure S3).

### 3.2 Protein Profiling of Bulk, Neuronal, Microglial, and Astrocytic EVs

Next, we conducted a comparative proteomic analysis of bulk and cell type‐specific EV populations. To evaluate proteins arising from non‐specific binding during immunocapture, an isotype IgG control was processed using the same workflow. A total of 4287 proteins were retained for downstream analyses (see Material and Methods). The IgG isotype control yielded only 118 proteins, with minimal overlap with proteins identified in the bulk and cell type‐specific EV samples. Therefore, no additional exclusion based on the control proteome was applied. The complete list of proteins detected in the IgG control is provided in Table S1.

**FIGURE 2:**
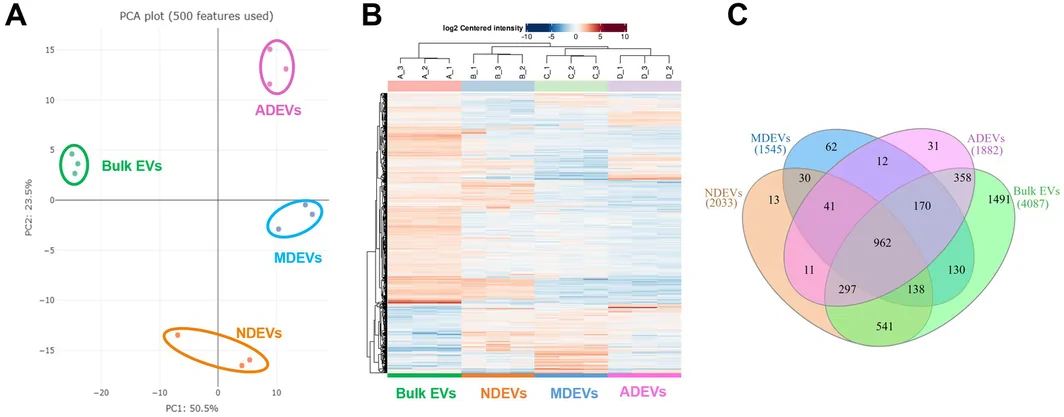
Measurement of proteins that provide separation among cell type‐specific and bulk EVs in the brain. (A) Principal component analysis (PCA) based on the top 500 most variable proteins reveals clear clustering by EV subpopulation group, indicating distinct proteomic profiles among NDEVs, MDEVs, ADEVs, and bulk EVs. (B) Heatmap of log_2_‐centered protein intensity values showing distinct expression patterns specific to each cell type‐specific EVs, and high reproducibility across biological replicates (*N* = 3 per group). (C) Venn diagram illustrating the distribution of proteins identified across NDEVs, MDEVs, ADEVs, and bulk EVs. This diagram was performed based on MaxLFQ Intensity.

Principal component analysis (PCA) revealed distinct sample distributions among EV subtypes, with segregated clustering patterns and high reproducibility across biological replicates (Figure 2A). A heatmap of log_2_‐centered protein intensities further illustrated different protein expression profiles among NDEVs, MDEVs, ADEVs, and bulk EVs, with high similarity in protein profiles observed within replicates (Figure 2B).

Proteomic analysis identified 4087 proteins in bulk EVs, while 2033 were detected in NDEVs, 1545 in MDEVs, and 1882 in ADEVs. A core set of 962 proteins, including canonical EV proteins such as ALIX, CD9, CD81, members of the Annexin family, and FLOT1, were shared across all EV groups (Figure 2C, Table S2). Notably, only 90 of these 962 shared proteins were also detected in the IgG control (Table S3). This limited overlap suggests that non‐specific binding contributed minimally to the common EV proteome. Exclusive proteins accounted for 1491 entries in bulk EVs, representing approximately 35% of all proteins. Among cell type‐specific EVs, 13 proteins were unique to NDEVs (e.g., VAMP7 and SLC7A4), 62 to MDEVs (e.g., GPR34, CLEC5A, and CLEC4A2), and 31 to ADEVs (e.g., LPR4 and KCNJ16). The complete list of proteins can be found in Table S2. Overlap analysis revealed that NDEVs shared the highest number of proteins with bulk EVs (1938, of which 541 were uniquely in NDEVs), including AMPA receptor subunits GRIA1, GRIA2, and GRIA3. By contrast MDEVs exhibited the most distinct profile, sharing 1400 proteins with bulk EVs and containing 130 unique proteins including the microglial markers CX3CR1 and TMEM119 (Figure 2C). These findings indicate that bulk EVs are predominantly neuronal in origin, while microglial EVs are the most underrepresented population.

### 3.3 Differential Protein Expression Profile in Cell Type‐Specific EVs

To further characterize the EV subtypes, differential expression analyses were performed among bulk EVs and cell type‐specific EVs using thresholds of Log_2_ fold‐change (FC) ≥ 1 and an adjusted *p* value < 0.05.

**FIGURE 3:**
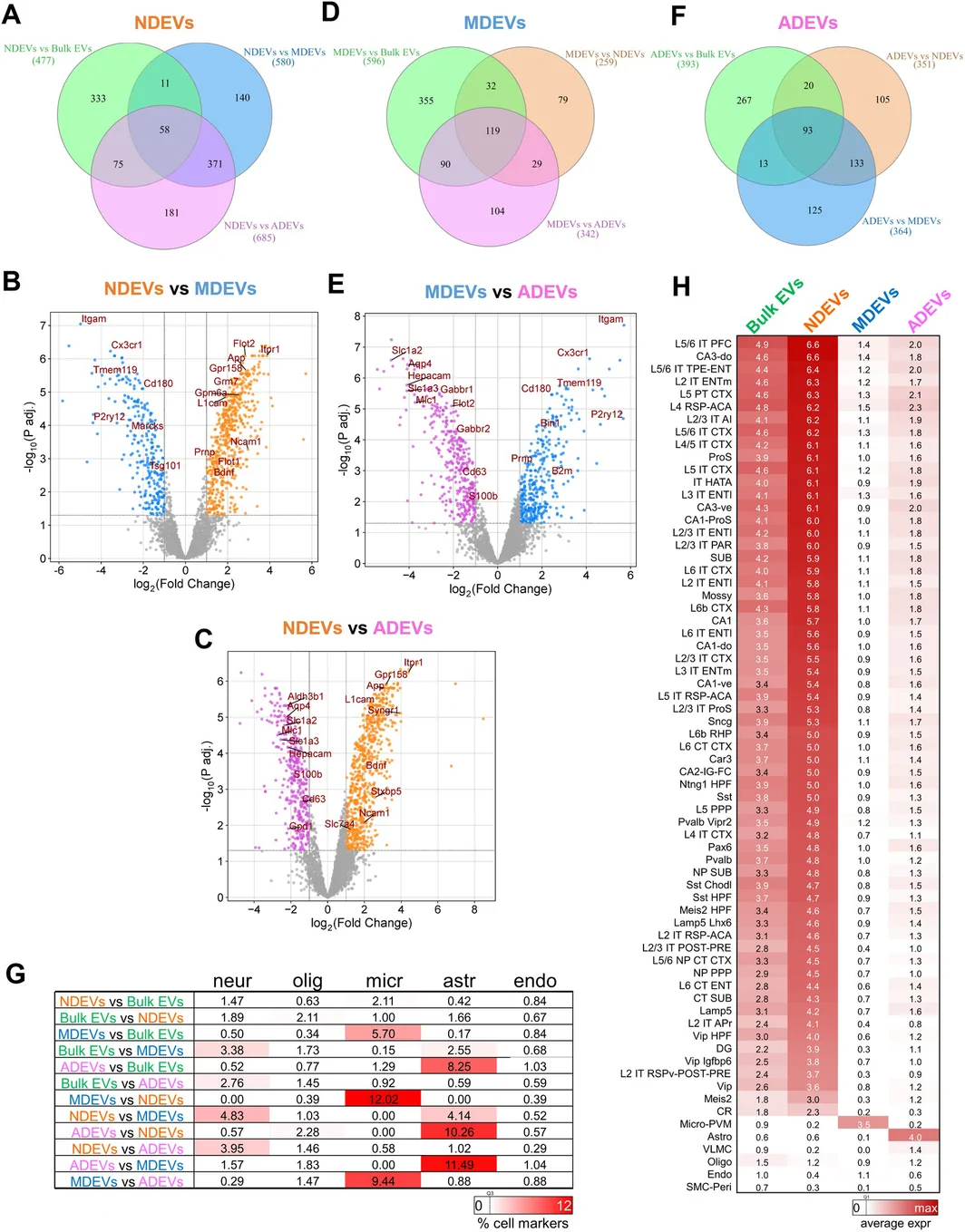
Differential protein signatures across cell type‐specific and bulk EVs. (A) Venn diagram illustrating proteins upregulated in NDEVs relative to bulk EVs and other cell type‐specific EV subtypes, revealing 58 uniquely upregulated proteins. (B) Volcano Plot comparing the upregulated proteins in NDEVs compared to MDEVs, showing the upregulated proteins in NDEVs versus MDEVs (GPR158, GRM7, ITPR1, APP, GPM6A, L1CAM, NCAM1, PRNP, FLOT1, and BDNF) and upregulated in MDEVs compared to NDEVs (CD11b (Itgam), CX3CR1, TMEM119, CD180, P2RY12, MARCKs, and TSG101). (C) Volcano Plot comparing the upregulated proteins in NDEVs compared to ADEVs, showing the upregulated proteins in NDEVs versus ADEVs (GPR158, ITPR1, APP, L1CAM, SYNGR1, BDNF, STXBP5, NCAM1, and SLC7A4) and upregulated in ADEVs compared to NDEVs (ALDH3B1, AQP4, SLC1A2, MLC1, SLC1A3, HEPACAM, S100B, CD63, and GPD1). *Note:* GRM7 was also upregulated in NDEVs relative to ADEVs but it is not shown because its parameters were nearly identical to APP, causing an overlap. (D) Venn diagram showing proteins upregulated in MDEVs compared with the remaining EV subtypes and bulk EVs, identifying 119 upregulated proteins. (E) Volcano Plot comparing the upregulated proteins in MDEVs compared to ADEVs, showing the upregulated proteins in MDEVs versus ADEVs (CD11b (Itgam), CX3CR1, TMEM119, CD180, P2RY12, BIN2, PRNP, and B2M) and upregulated in ADEVs compared to MDEVs (SLC1A2, AQP4, HEPACAM, SLC1A3, MLC1, FLOT2, GABBR1‐2, CD63, and S100B). (F) Venn diagram depicting the proteins upregulated in ADEVs, with 93 proteins elevated in this cell type‐specific EV population. (G) Heatmap displaying the percentage of brain cell‐specific markers described from a transcriptomics meta‐analysis. neur = neuron; olig = oligodendrocyte; micr = microglia; astr = astrocyte; endo = endothelial cells. (H) Heatmap showing the average expression of upregulated NDEVs, MDEVs or ADEVs proteins in different cell‐type clusters defined by scRNA‐seq (Allen brain atlas). Volcano plots show gene names instead of protein names.

NDEVs exhibited 58 proteins exclusively upregulated compared to all other groups, including BDNF, GRM7, ITPR1, and GPR158 (Figure 3A–C, Table S4). GO Biological Process enrichment analysis revealed that these proteins were primarily involved in localization, transport, transmembrane and ion transport, and cell–cell signaling (Figure S4). In pairwise comparisons, NDEVs showed 371 proteins upregulated relative to MDEVs and ADEVs, including the well‐known NDEV markers NCAM1 and L1CAM, as well as neurodegeneration‐associated proteins such as APP (Figure 3A–C). Additionally, 181 were elevated relative to ADEVs (e.g., SYNGR1 and STXBP5; Figure 3A,C) and 140 proteins versus MDEVs (e.g., GPM6A, FLOT1; Figure 3A,B). Notably, 11 proteins were uniquely upregulated in NDEVs compared with both bulk EVs and MDEVs (e.g., FLOT2; Figure 3A,B), and 75 proteins were shared as upregulated relative to bulk EVs and ADEVs (e.g., SLC7A4; Figure 3A,C; Table S4).

MDEVs contained 119 proteins upregulated compared to bulk EVs and other cell type‐specific EVs, which included the microglial markers ITGAM, CX3CR1, TMEM119, and P2RY12 (Figure 3B,D,E). These proteins were enriched in pathways related to cell activation, immune processes, and vesicle‐mediated transport (Figure S4). Pairwise comparisons identified 79 proteins elevated versus NDEVs (e.g., TSG101 and MARCKS; Figure 3B,D) and 104 versus ADEVs (e.g., PRNP, B2M, BIN1; Figure 3D,E), with 29 proteins consistently increased in MDEVs compared with both NDEVs and ADEVs (e.g., CD180; Figure 3C–E, Table S5).

ADEVs displayed 93 proteins upregulated relative to bulk EVs, NDEVs and MDEVs, including the astroglial markers AQP4, S100B, EAAT2, and GlialCAM (HEPACAM) (Figure 3C,E,F). GO analysis indicated enrichment for amino acid and organic acid transport, as well as chemical homeostasis (Figure S4). In pairwise comparisons, 133 proteins were upregulated versus NDEVs and MDEVs, including EAAT1, CD63, and MLC1 (Figure 3C,E,F). Specifically, 125 proteins were elevated versus MDEVs (e.g., GABBR1‐2 and FLOT2; Figure 3E,F) and 105 versus NDEVs (e.g., GPD1‐2, ALDH3B1; Figure 3C,F; Table S6).

To further validate cell type‐specificity of the retrieved EV proteins, we examined the presence of curated top 100 cell‐specific expressed genes that were described for each major brain cell type (McKenzie et al. 2018) across the pairwise comparisons. ADEVs and MDEVs showed strong overlap with reported markers, whereas NDEV enrichment was more moderate (Figure 3G), consistent with their similarity to bulk EVs. In contrast, integration with single‐cell RNAseq data from the Allen Brain Atlas (see Material and Methods), confirmed representation of major neuronal‐cell types and brain regions in our NDEVs proteome (Figure 3H), that is, no apparent enrichment for specific neuronal subtypes was observed; this analysis also supported the specificity of MDEV and ADEV profiles (Figure 3H). Notably, differentially expressed proteins in bulk EVs were mainly of neuronal origin, although their averaged gene expression was lower compared to NDEV corresponding genes, with poor representation of oligodendrocyte, endothelial, and pericyte gene markers (Figure 3H).

### 3.4 Distribution of the Commonly Identified EV Markers Across Cell Type‐Specific EVs

**FIGURE 4:**
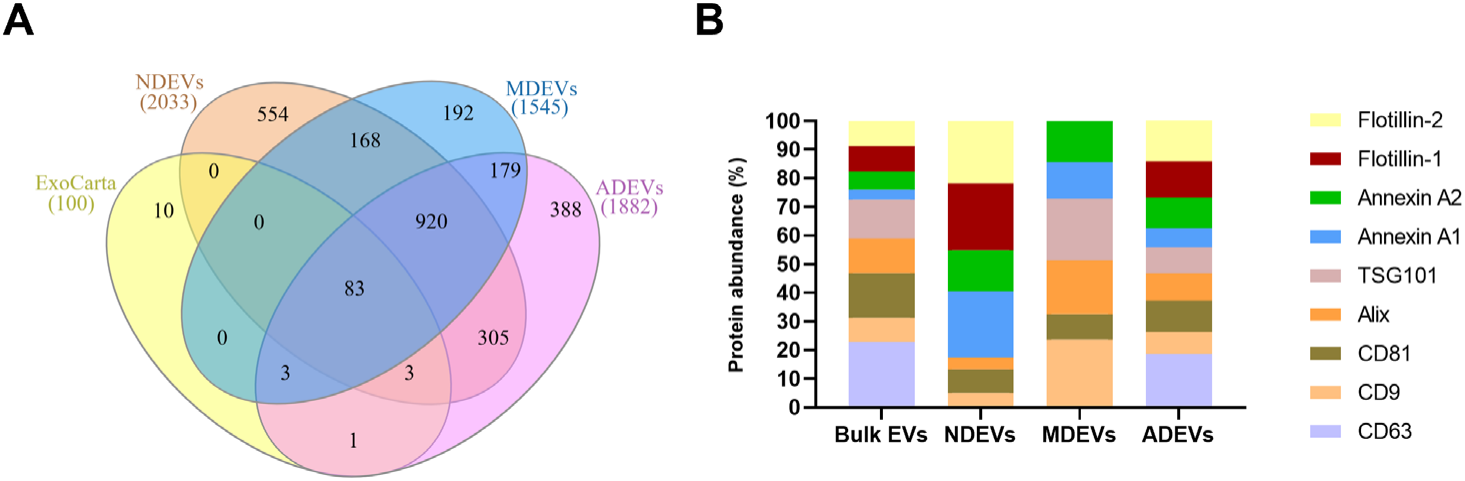
Distribution of commonly identified EV markers across bulk and cell type‐specific EVs. (A) Venn diagram showing the overlap between proteins identified in NDEVs, ADEVs, MDEVs, and the ExoCarta Top 100 small EV protein list. A total of 83 proteins were shared between the cell type‐specific EV datasets and the ExoCarta reference list. (B) Stacked bar chart showing the protein abundance (%) of common EV markers across cell type‐specific EVs and bulk EVs. The protein abundance was normalized to total MaxLFQ intensity.

To characterize the distribution of known canonical EV markers among the different EV populations, we compared our detected proteins with the ExoCarta Top 100 small EV protein list (http://exocarta.org/sEV_top100). Of the 100 catalogued proteins, 83 were identified in all our datasets. Common markers included Annexins (ANXA1, ANXA2, ANXA5, ANXA6), tetraspanins (CD9, CD81), and the Endosomal Sorting Complex Required for Transport (ESCRT)‐accessory protein ALIX. Three proteins (FLOT2, TFRC, MYO1C) were shared between NDEVs and ADEVs, three (RDX, TSG101, EHD1) overlapped between MDEVs and ADEVs, and one (CD63) was detected only in ADEVs (Figure 4A; Table S6).

Flotillin family members (FLOT1, FLOT2) were significantly enriched in NDEVs (*p* adj = 1.86 × 10^−3^; 8.14 × 10^−7^) and ADEVs (*p* adj = 9.96 × 10^−3^; 8.06 × 10^−6^) compared to MDEVs. NDEVs also showed higher FLOT2 levels relative to bulk EVs (*p* adj = 6.18 × 10^−4^). Among Annexins, ANXA1 was elevated in NDEVs but not significantly different from ADEVs or MDEVs, while it was significantly enriched in bulk EVs compared to NDEVs and MDEVs (*p* adj = 1.09 × 10^−2^ and 2.05 × 10^−2^). ANXA2 was consistently expressed across EV subtypes but significantly higher in all cell type–specific EVs compared to bulk EVs (vs. NDEVs *p* adj = 3.90 × 10^−3^; vs. MDEVs *p* adj = 2.16 × 10^−3^; vs. ADEVs *p* adj = 0.0191).

Regarding tetraspanins, CD9 was markedly enriched in MDEVs relative to NDEVs (*p* adj = 2.66 × 10^−5^) and ADEVs (*p* adj = 9.89 × 10^−4^), as well as bulk EVs (*p* adj = 1.65 × 10^−3^). CD63 was only detected in ADEVs and bulk EVs, with no significant difference between them (*p* adj = 0.234). CD81 expression remained relatively stable but was higher in bulk EVs than in cell type–specific EVs (vs. NDEVs *p* adj = 7.36 × 10^−4^; vs. MDEVs *p* adj = 7.92 × 10^−4^; vs. ADEVs *p* adj = 0.0141).

Finally, ESCRT‐related proteins ALIX and TSG101 were significantly enriched in MDEVs compared to NDEVs (*p* adj = 0.00129 and 0.00234, respectively), with ALIX also elevated in ADEVs relative to NDEVs (*p* adj = 2.34 × 10^−2^). In contrast, MDEVs showed no significant enrichment relative to bulk EVs for these proteins. Table 1 summarizes fold‐changes and *p* adj values for the expression of common EV markers across cell type‐specific EVs. The relative abundance of each EV marker across EV populations is shown in Figure 4B.

**TABLE 1:** Enrichment patterns of common EV markers in bulk and cell type‐specific EVs.

A—Common EV markers distributions across cell type‐specific EVs							
Protein name	Gene name	log_2_ FC	log_2_ FC	log_2_ FC	*p* adj	*p* adj	*p* adj
		NDEVs vs. MDEVs	NDEVs vs. ADEVs	MDEVs vs. ADEVs	NDEVs vs. MDEVs	NDEVs vs. ADEVs	MDEVs vs. ADEVs
ANXA1	Anxa 1	1.17	0.346	0.825	0.127	0.756	0.303
ANXA2	Anxa2	−0.02	0.282	0.302	0.971	0.33	0.316
CD63	Cd63	0.175	−1.44	−1.62	7.74E−01	2.18E‐03	1.70E‐03
CD81	Cd81	−0.128	−0.533	−0.405	0.752	0.0609	0.17
CD9	Cd9	−2.24	−0.77	1.47	2.66E‐05	0.0343	9.89E‐04
FLOT1	Flot1	2.62	0.486	−2.13	1.86E‐03	0.118	9.96E‐03
FLOT2	Flot2	2.84	0.758	−2.08	8.14E‐07	6.36E‐03	8.06E‐06
ALIX	Pdcd6ip	−2.29	−1.41	0.879	0.00129	0.0234	0.164
TSG101	Tsg101	−1.76	−0.813	0.946	0.00234	0.103	0.0772

B—Common EV markers distributions between bulk EVs and cell type‐specific EVs							
Protein name	Gene name	log_2_ FC	log_2_ FC	log_2_ FC	*p* adj	*p* adj	*p* adj
		Bulk EVs vs. NDEVs	Bulk EVs vs. MDEVs	Bulk EVs vs. ADEVs	Bulk EVs vs. NDEVs	Bulk EVs vs. MDEVs	Bulk EVs vs. ADEVs
ANXA1	Anxa 1	−2.01	−1.66	−0.837	1.09E−02	2.05E−02	0.204
ANXA2	Anxa2	−0.916	−0.936	−0.634	3.90E‐03	2.16E‐03	0.0191
CD63	Cd63	1.86	2.04	0.42	5.11E‐04	1.27E‐04	0.234
CD81	Cd81	1.19	1.06	0.653	7.36E‐04	7.92E‐04	0.0141
CD9	Cd9	1.05	−1.18	0.286	5.25E‐03	1.65E‐03	0.338
FLOT1	Flot1	−1.03	1.59	−0.541	0.118	1.81E‐02	0.379
FLOT2	Flot2	−1.11	1.73	−0.355	6.18E−04	1.21E−05	0.11
ALIX	Pdcd6ip	1.93	−0.363	0.516	3.56E‐03	0.475	0.31
TSG101	Tsg101	1.36	−0.395	0.551	8.61E‐03	0.351	0.198

*Note:* Log_2_ fold changes (FC) and adjusted *p* values (*p* adj) are shown.

### 3.5 G Protein‐Coupled Receptors (GPCRs) in Cell Type‐Specific EVs

**FIGURE 5:**
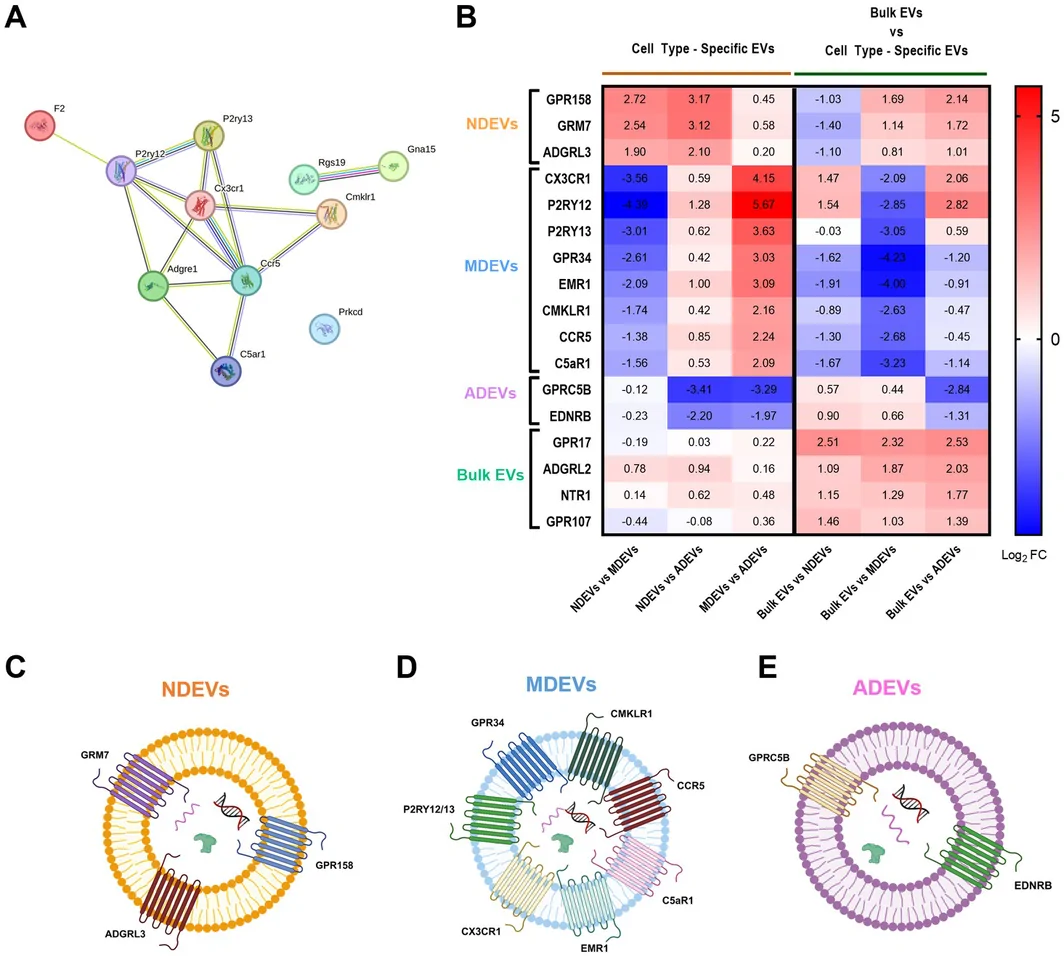
Differential enrichment of G protein‐coupled receptors (GPCRs) in cell type‐specific EVs and bulk EVs. (A) MDEVs signaling by GPCR pathway in Reactome Pathways from the up‐regulated proteins in MDEVs (FC ≥ 1 and *p* adj < 0.05). (B) A heatmap displaying the Log_2_ Fold Change (Log_2_ FC) of GPCRs significantly enriched in each cell type‐specific EV compared to the Bulk EVs. The top‐enriched receptors are GPR158 in NDEVs, CX3CR1 in MDEVs, and GPRC5B in ADEVs. GPR17 is significantly enriched in Bulk EVs. (C–E) Graphical illustration of the most enriched GPCRs by cell type‐specific EVs (created on Biorender.com). MDEVs Reactome Pathways show gene names instead of protein names.

GPCRs are key regulators of neurotransmission, synaptic plasticity, neuroinflammation, and neuromodulation, and their presence in EVs suggests that cells may export signaling receptors to modulate the extracellular environment (Bebelman et al. 2020; Di Niro et al. 2024). Reactome pathway analysis identified GPCR signaling as one of the most significantly enriched pathways in MDEVs (Figure 5A), consistent with the broad enrichment of GPCR‐related proteins observed in the proteomic analysis.

To further investigate GPCR representation across EV subtypes, we compared proteins upregulated in each EV population (FC ≥ 1, adjusted *p* < 0.05) against curated GPCR lists from the IUPHAR/BPS Guide to PHARMACOLOGY (https://www.guidetopharmacology.org/GRAC/ReceptorFamiliesForward?type=GPCR). Although GPCR‐related GO terms were not significantly enriched in NDEVs or ADEVs, this exploratory analysis revealed distinct GPCR signatures across EV subtypes.

NDEVs were characterized by enrichment of GPR158, which was significantly increased relative to bulk EVs (*p* adj = 0.00214), MDEVs (*p* adj = 2.72 × 10^−6^), and ADEVs (*p* adj = 1.27 × 10^−6^) (Figure 5B,C). Additional GPCRs enriched in NDEVs included the adhesion receptor ADGRL3 and the metabotropic glutamate receptor GRM7.

MDEVs displayed strong enrichment of immune‐ and microglia‐associated GPCRs, including the chemokine receptors CX3CR1 and CCR5, the purinergic receptors P2RY12 and P2RY13, the chemerin receptor CMKLR1, the complement receptor C5aR1, and the orphan receptor GPR34 (Figure 5B–D). Among these, CX3CR1 showed the strongest enrichment compared with all other EV populations (vs. bulk EVs *p* adj = 7.91 × 10^−6^; vs. NDEVs *p* adj = 5.88 × 10^−7^; vs. ADEVs *p* adj = 2.40 × 10^−7^), while P2RY12, P2RY13, and GPR34 were also elevated relative to bulk EVs, NDEVs, and ADEVs.

ADEVs selectively enriched the Class C orphan receptor GPRC5B together with the endothelin receptor EDNRB (Figure 5B–C). GPRC5B represented the most significantly enriched GPCR in ADEVs compared with all other EV populations (vs. bulk EVs *p* adj = 3.10 × 10^−6^; vs. NDEVs *p* adj = 1.57 × 10^−6^; vs. MDEVs *p* adj = 2.46 × 10^−6^).

In contrast, bulk EVs preferentially enriched GPR17, a Class A P2Y‐like purinergic receptor, relative to NDEVs (*p* adj = 1.59 × 10^−4^), MDEVs (*p* adj = 1.16 × 10^−4^), and ADEVs (*p* adj = 6.57 × 10^−5^). Additional GPCRs enriched in bulk EVs included ADGRL2 (Latrophilin‐2), NTR1, and the orphan receptor GPR107 (Figure 5B).

### 3.6 ADEVs Harbor a Proteomic Signature Linked to BBB Function via the GlialCAM Interactome

Astrocytes are crucial regulators of CNS homeostasis, BBB integrity, and their dysfunction is strongly associated with neurodegenerative diseases such as Alzheimer's, Parkinson's, and Amyotrophic Lateral Sclerosis. Given their central role in neurovascular regulation and glia–neuron communication, we aimed to identify molecular signatures within ADEVs associated with astrocyte endfeet and BBB interfaces.

To define an ADEV‐specific signature, we first identified proteins consistently upregulated in ADEVs compared to bulk EVs, NDEVs and MDEVs, or only to NDEVs and MDEVs. This initial overlap analysis yielded 226 proteins (Figure S5). Applying a stringent threshold (FC ≥ 2 and *p* adj < 0.05) reduced this set to 66 highly upregulated proteins (Figure S5B): 11 proteins upregulated against both bulk EVs and other cell type‐specific EVs, and 55 proteins upregulated against cell type‐specific EVs only.

STRING‐based PPI analysis and k‐means clustering (*k* = 7) revealed a major cluster related to Glial cell projection, including established ADEV markers EAAT1, EAAT2, GLUL, and AQP4 (Figure S5C). This cluster also contained GlialCAM‐associated proteins such as GlialCAM, MLC1, GPR37L1, TTYH1, and GJA1. Other clusters included lysosphingolipid and LPA receptors (S1PR1, LPAR1, PLPP3, SPTLC1), modulator molecules of the bioactive lipid receptors activity (RGS6, ARHGEF12), postsynaptic actin remodeling (WASF3, WASF1), and adenosylhomocysteinase activity (AHCYL1, AHCYL2) (Figure S5C).

GPRC5B and TSPAN9, which are members of the GlialCAM interactome, were also upregulated in ADEVs but clustered outside the main Glial cell projection group. Specifically, GPRC5B clustered with NUDT3 (Figure S5C), suggesting a functional interaction based on co‐expression evidence. ATP1B2, a known astrocyte marker, was excluded from this stringent set due to FC values slightly below threshold versus bulk EVs (FC = −1.17) and NDEVs (FC = −1.34).

**FIGURE 6:**
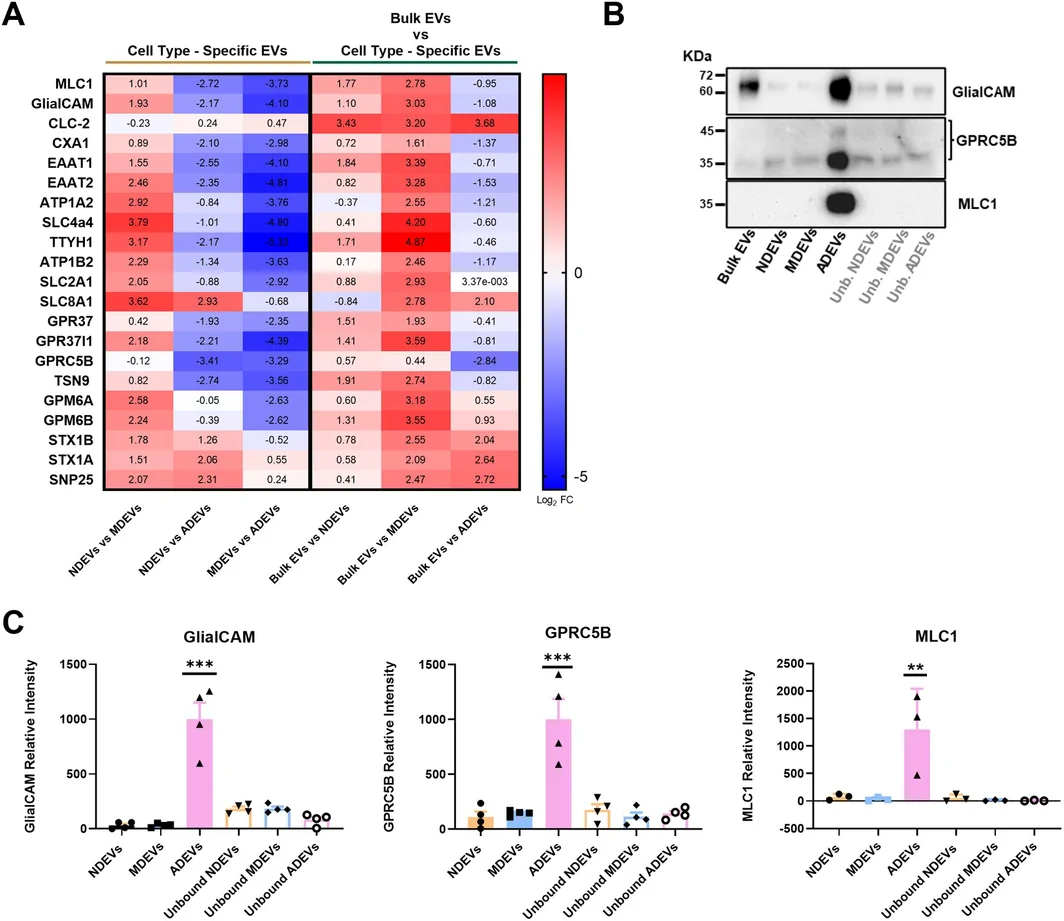
Proteomic profiling and experimental validation of the GlialCAM interactome signature in ADEVs. (A) Heatmap illustrating the relative enrichment of GlialCAM interactome proteins in ADEVs, NDEVs, and MDEVs compared to bulk EVs and/or other cell type‐specific EVs. This analysis highlights the cell type‐specific distribution of astrocytic (e.g., MLC1, GlialCAM) versus neuronal (e.g., STX1A, SNAP25) markers within the interactome. (B) Western blot validation of key astrocytic GlialCAM interactome proteins in ADEVs. Immunoblotting confirms the enrichment of the astrocytic markers MLC1, GlialCAM, and GPRC5B in ADEVs compared to other cell type‐specific EVs (NDEVs and MDEVs). (C) Bar graphs quantifying the relative abundance (mean ± SD) of GlialCAM, GPRC5B and MLC1, in the bound fractions (ADEVs, MDEVs, and NDEVs) versus the unbound (Unb.). Data is normalized by the average intensity in ADEVs. Ordinary one‐way ANOVA followed by Tukey's multiple comparisons test, *N* = 4 for GlialCAM and GPRC5B (***p < 0.0001); *N* = 3 for MLC1 (**p < 0.005).

Analysis of GlialCAM interactome membership confirmed supported cell type‐specificity in protein subtype‐specific enrichment: ADEVs were enriched for adhesion and transport proteins (e.g., EAAT1‐2, MLC1), while NDEVs showed enrichment for synaptic junctions within the same interactome (e.g., STX1A, STX1B, SNAP25; FC vs. ADEVs = 2.06, 1.26 and 2.31, respectively). GPM6A and GPM6B were similarly abundant in both ADEVs and NDEVs (Figure 6A).

Western blot validated the significant enrichment of the three key GlialCAM interactome components in ADEVs: MLC1, GlialCAM, and GPRC5B (Figure 6B,C). These findings suggest that ADEVs act as carriers of molecular machinery essential for BBB support. Specifically, MLC1 and GlialCAM maintain astrocyte endfeet structure and BBB integrity, while GPRC5B enrichment indicates potential roles in receptor‐mediated communication pathways for brain homeostasis.

## 4 Discussion

EVs secreted by brain cells represent a key interface for neuro‐glial communication and a promising source of biomarkers for CNS disorders. By comprehensively characterizing neuron‐, microglia‐, and astrocyte‐derived EVs (NDEVs, MDEVs, and ADEVs) from mouse brain tissue, we delineate a proteomic landscape that captures the molecular diversity of brain EVs.

Each EV subtype exhibited molecular fingerprints consistent with parental cell functions. NDEVs were enriched in synaptic and vesicle trafficking proteins such as APP, SNAP25, L1CAM, NCAM1, and BDNF, reflecting roles in cell adhesion, neurotransmission, and synaptic plasticity. Although bulk EVs were highly similar to NDEVs, our data indicate that multiple neuronal subtypes (both excitatory and inhibitory) from diverse brain regions contribute to the NDEV pool. To explain the similarity between bulk EVs and NDEVs, we can think that neurons release the vast majority of EVs in the brain, challenging to reach saturating conditions in the immunocapture; alternatively, NDEVs are highly heterogeneous and express distinct surface markers. In the first case, this will explain why bulk EVs were not enriched with oligodendrocytic EVs, and in the latter case, this will justify the apparent controversy regarding the use of different markers to pull down NDEVs in the literature. MDEVs contained immune and phagocytic markers including bona fide microglial markers such as CX3CR1, P2RY12/13, and CCR5, consistent with their functions in neuroimmune signaling and debris clearance. ADEVs were characterized by astrocytic transporters and endfoot‐associated proteins such as EAAT1/2, AQP4, and GJA1, consistent with a proteomic signature linked to neurotransmitter recycling, metabolic homeostasis, and BBB function. Overall, these findings align with previous reports demonstrating distinct proteomic profiles of neuron‐, microglia‐, and astrocyte‐derived EVs in human iPSC‐derived brain cells (You et al. 2022), and human brain tissue where neuronal (SNAP25, GRM7, GPR158) and astroglial markers (AQP4, EAAT1/GLAST) have been detected in total EV preparations (Espourteille et al. 2025; Nussbaumer et al. 2025) and linked to neurodegenerative pathology.

Comparison with the ExoCarta Top 100 small EV proteins confirmed shared EV architecture among subtypes, yet revealed cell type‐dependent distribution: flotillins (FLOT1, FLOT2) were enriched in NDEVs and ADEVs, suggesting flotillin‐positive membrane microdomains as EV origins (Otto and Nichols 2011; Phuyal et al. 2014), whereas MDEVs showed higher levels of ALIX and TSG101, indicating a stronger reliance on ESCRT‐dependent endosomal biogenesis (Colombo et al. 2013). These differences support the existence of multiple EV biogenesis routes across brain cell types that shape their molecular cargo and signaling functions and should be taken into consideration when selecting EV markers for characterization and validation of brain cell‐specific EVs.

A striking observation was the representation of GPCRs across EVs, underscoring the importance of receptor‐mediated signaling in EV biology. NDEVs were enriched in class C receptors including the metabotropic glutamate receptor family and GPR158, linked to synaptic signaling and cognitive regulation (Khrimian et al. 2017; Verpoort et al. 2025; Wei et al. 2024); MDEVs contained chemokine and purinergic GPCRs such as CX3CR1, GPR34, P2RY12, and P2RY13, consistent with their immune‐sensing roles (Etani et al. 2025; Hsiao et al. 2021; Schöneberg et al. 2018; Škandík et al. 2025; Zhu et al. 2026); and ADEVs exhibited selective enrichment of the astroglial orphan receptors GPR37L1 and GPRC5B, both implicated in glial stress responses and neuroprotection (Alonso‐Gardón et al. 2021; Meyer et al. 2013). The presence of GPCRs in EVs suggests that they may act as carriers of functional receptors, modulating intercellular communication and potentially participating in feedback regulation of signaling pathways (Bebelman et al. 2020; Di Niro et al. 2024; Kwon et al. 2014). Importantly, EV‐associated GPCRs (EV‐GPCRs) have been linked to various pathological contexts (Bebelman et al. 2020; Kahn et al. 2017; Kwon et al. 2014; Pironti et al. 2015), including Alzheimer's and Tau pathologies (Sinsky et al. 2020; Zhang, Hamit, et al. 2025), positioning them as promising biomarker candidates for neurodegenerative disorders.

In this study, ADEVs were immunocaptured using the ACSA‐2 antibody, which recognizes ATP1B2. Although ATP1B2 is not exclusively expressed by astrocytes, previous studies have shown that it is significantly enriched in astrocytes compared to other brain cell types, supporting its use as a marker for ADEVs (Batiuk et al. 2017; Hou et al. 2022). Consistent with this, ATP1B2 has previously been used for the isolation of ADEVs from human blood samples, further supporting its suitability for ADEV enrichment (Zhang, Lobb, et al. 2025). In addition, ATP1B2 is enriched in astrocytic endfeet and has been associated with proteins relevant to the BBB (Hill et al. 2025), which aligns with the biological pathways highlighted in our study.

Within ADEVs, we identified a coherent network of GlialCAM‐associated proteins, including MLC1, GlialCAM, ATP1B2, EAAT1/2, TTYH1, GJA1, GPRC5B, and GPR37L1, representing a proteomic signature of astrocytic endfeet and proteins known to localize to the neurovascular unit (Alonso‐Gardón et al. 2021; Hill et al. 2025). Supporting our results, GlialCAM and GPRC5B were also found in ADEVs from in vitro human IPSC‐derived cultures (You et al. 2022). Furthermore, this observation converges with recent studies showing that astroglial exosomal GlialCAM mediates axonal growth and neuroprotection, while inflammatory cytokines reduce GlialCAM‐positive EV release in mouse models of amyotrophic lateral sclerosis (Jin et al. 2024). The detection of GPRC5B in ADEVs aligns with its previously reported exosomal sorting and intercellular transfer (Kwon et al. 2014, 2016), suggesting that GPCRs within the GlialCAM interactome may be transported by EVs. The overlap between the ADEV proteome and the GlialCAM/MLC1 interactome also links EV composition to ion and water homeostasis pathways implicated in megalencephalic leukoencephalopathy with subcortical cysts (MLC), supporting the hypothesis that ADEVs could reflect functional states of astrocytic endfeet relevant to BBB integrity and white‐matter homeostasis. Notably, GPRC5B and GPR37L1, validated components of the GlialCAM interactome, modulate MLC1 and GlialCAM levels, and GPRC5B has been associated with MLC genetics (Alonso‐Gardón et al. 2021), reinforcing its translational interest. Several members of this network, including EAAT1 (Valle‐Tamayo et al. 2022; You et al. 2022), EAAT2, and ATP1B2 (Zhang, Lobb, et al. 2025), are cell‐surface proteins already used for ADEV isolation, highlighting their immuno‐targeting potential. In contrast, although GlialCAM has been detected in EVs (Jin et al. 2023, 2024), it has never been used directly as an immunoprecipitation target. MLC1, while less studied in the EV field, is listed in EV proteome databases (Chitti et al. 2024) and localizes to membrane, endosomal, and multivesicular body (MVB) compartments in astrocytes (Lanciotti et al. 2010), which makes it a candidate for future validation as an EV‐associated protein or immunocapture target.

Integrating the ADEVs proteome with GPCR and canonical EV marker profiles further suggests a complex interplay between vesicle biogenesis pathways, signaling functions, and neurovascular specialization. Additional members of the GlialCAM‐associated GPCR family, such as GPR37L1, have been identified as EV‐associated proteins in EV‐enriched populations spontaneously released from mouse and human brain tissue (Gomes et al. 2023), and GPRC5B has similarly been detected in EVs across multiple studies (Kwon et al. 2014, 2016; You et al. 2022). Our results, together with these previously reported associations of GlialCAM‐linked GPCRs with EVs, further support their conserved localization within ADEV populations.

While our dataset provides a robust reference map for brain cell–specific EVs, some limitations should be acknowledged. The use of young adult brain tissue may underestimate age‐related or pathology‐specific alterations. Moreover, regional and subtype heterogeneity within astrocyte and microglial populations could contribute to undetected EV diversity. We also note that GPM6A, previously reported as a neuronal EV marker (Espourteille et al. 2025), was enriched in both NDEVs and ADEVs, potentially reflecting overlapping molecular characteristics between these vesicle populations. In addition, it should be noted that the isolation of tissue‐derived EVs presents unique challenges as compared to EVs from biofluids or conditioned media, as tissue dissociation is required through mechanical and enzymatic dissociation procedures that can potentially introduce artifacts, including cellular disruption and alterations to EV‐associated proteins. We selected papain as the dissociation enzyme because it dissociates brain tissue while minimizing intracellular contamination and preserving EV integrity (D'Acunzo et al. 2022, 2021; Pérez‐González et al. 2025; Polanco et al. 2016), and it has been widely used for neural tissue preparation (Brewer and Torricelli 2007). Nevertheless, as with any enzymatic dissociation method, papain may affect the detection of certain membrane‐associated proteins through proteolytic cleavage of extracellular domains. Consequently, it cannot be ruled out that the recovery of some EV surface markers was influenced by the tissue dissociation procedure. In this line, although comprehensive characterization by Western blotting, NTA, and EM confirmed EV enrichment in our preparations, we cannot completely exclude the possibility that some proteins identified in the GlialCAM interactome originated from astrocytic membrane fragments or disrupted perivascular astrocytic endfeet co‐isolated during tissue processing. Therefore, the observed enrichment of GlialCAM‐associated proteins may, to some extent, reflect contributions from these structures in addition to bona fide EV cargo. However, studies in vitro have shown the presence of some of these proteins in ADEVs, reinforcing their nature as EV components (Jin et al. 2024). Finally, we need to acknowledge that this study is primarily descriptive. Future investigations incorporating functional analyses of ADEVs and relevant disease models will be essential to determine how ADEVs interact with other components of the neurovascular unit and how their molecular composition changes in response to BBB dysfunction and neurodegenerative disease progression.

In summary, our comparative proteomic profiling establishes a detailed atlas of neuron‐, microglia‐, and astrocyte‐derived EVs, offering a valuable resource for target selection and cargo characterization. Furthermore, we report that ADEVs exhibit a distinctive molecular signature enriched in BBB‐associated proteins and GPCRs that suggest that these vesicles capture molecular features of astrocytic pathways involved in neurovascular biology. Together, these findings provide a molecular foundation for exploring cell type‐specific biomarkers in EVs and for investigating how ADEV proteomic signatures relate to astrocytic endfoot biology, BBB‐associated pathways, and neurodegenerative disorders.

## Author Contributions

Material preparation and data collection were performed by Alba M. Lucart‐Sanchez, Edward Sellés‐Climent, Jorge Navarro‐Calvo, Guillem Pont‐Espinós, Jose A. Gomez‐Sanchez, and Rocío Pérez‐González. Data analyses were performed by Alba M. Lucart‐Sanchez, Luis M. Valor, and Rocío Pérez‐González. Rocío Pérez‐González conceived the study. Raúl Estévez contributed to the conceptual development of the research. The first draft of the manuscript was written by Alba M. Lucart‐Sanchez and Rocío Pérez‐González, and all authors provided critical feedback on earlier versions of the manuscript. All authors read and approved the final manuscript.

## Funding

PID2022‐137426OA‐I00 funded by MICIU/AEI/10.13039/501100011033/ and by FSE+, and the EU Joint Programme‐ Neurodegenerative Diseases (JPND2022‐115, CP22/00050) ‐ EPI‐3E. R.P.G. is recipient of a Miguel Servet fellowship CP21/00009 from the Instituto de Salud Carlos III (ISCIII). Work in the Estévez lab was supported by PID2024‐159674NB‐100 funded by MICIU/AEI/10.13039/501100011033/ and by FEDER/UE. Dr. Valor's research is supported by CNS2022‐136169 funded by Ministerio de Ciencia e Innovación—NextGenerationEU 2021–2023, and PI23/01568 funded by ISCIII and Fondo Europeo de Desarrollo Regional 2014–2020. J.N.C. is recipient of a predoctoral fellowship from ISABIAL (2024/D/4).

## Ethics Statement

All mouse procedures were approved by the Comité de Ética de la Investigación, Universidad de Alicante, and authorized by the Dirección General de Producción Agrícola y Ganadera, Generalitat Valenciana, according to European, national, and regional laws.

## Conflicts of Interest

The authors declare no conflicts of interest.

## Supporting information


**Figure S1:** Experimental workflow for the isolation of bulk and cell type‐specific extracellular vesicles (EVs). Schematic overview of the experimental workflow used to isolate bulk EVs and immunocapture cell type‐specific EVs from mouse brain for proteomic characterization. Neuron‐ (NDEVs), microglia‐ (MDEVs), and astrocyte‐derived EVs (ADEVs).

**Figure S2:** Validation of bulk extracellular vesicle (EV) isolation. (A) Representative Western blots showing enrichment of the EV markers Alix and CD63, and depletion of the endoplasmic reticulum marker Calnexin, in bulk EVs compared with P300 and P10K fractions (*N* = 4). A total of 5 μg of protein was loaded per well. (B) Quantification of Western blot bands confirms lower Calnexin and higher Alix and CD63 levels in bulk EVs relative to P300 and P10K. CD63 levels were also higher in P10K than in P300. The intensity of the bands was normalized to the total protein load estimated by the imaged stain‐free membrane intensity. (C) Representative Western blots showing reduced levels of the nuclear marker Lamin A/C, oligodendrocyte marker MBP, mitochondrial markers VDAC and COX IV, and synaptic marker Synaptophysin in bulk EVs compared to P300 and P10K (*N* = 4). A total of 5 μg of protein was loaded per well. (D) Quantification of Western blot bands confirms depletion of Lamin A/C, MBP, VDAC, and COX IV in bulk EVs, together with reduced synaptophysin levels relative to P300 and P10K fractions. The intensity of the bands was normalized to the total protein load estimated by the imaged stain‐free gel intensity. Overall, these results demonstrate enrichment of EV‐associated markers and depletion of cellular contaminants in bulk EVs. Statistical analysis was performed using ordinary one‐way ANOVA followed by Tukey's multiple‐comparisons test. Data are presented as mean ± SD. *p < 0.05, **p < 0.01, ***p < 0.001. P300 = pellet after 300 × g centrifugation; P10K = pellet after 10,000 × g centrifugation; bulk EVs (P100K) = pellet after 100,000 × g centrifugation.

**Figure S3:** Extended electron microscopy images of bulk and cell type‐specific extracellular vesicles (EVs). Representative images showing EVs with expected size and morphology in bulk EVs, neuron‐derived EV (NDEV), microglia‐derived EV (MDEV), and astrocyte‐derived EV (ADEV) preparations. Images were acquired at 10 μm magnification. Scale bars (200 nm) are included in each image. Left panel of images correspond to a larger field view of the region in Figure 1 of the manuscript.

**Figure S4:** GO biological process of cell type‐specific EV subtypes. (A) GO Biological Process enrichment analysis of the 58 NDEV‐upregulated proteins, highlighting pathways related to establishment of localization, transmembrane and ion transport, and cell–cell signaling. (B) GO Biological Process analysis of the 119 MDEV‐upregulated proteins, demonstrating enrichment in cell activation, immune system processes, immune response, and vesicle‐mediated transport. (C) GO Biological Process enrichment of the 93 ADEV‐associated proteins, showing pathways related to amino acid transmembrane transport, carboxylic and organic acid transport, and chemical homeostasis.

**Figure S5:** Detailed proteomic network analysis of proteins upregulated in ADEVs. (A) Protein‐Protein Interaction (PPI) Network including the full set of 226 proteins identified as consistently upregulated in ADEVs (FC ≥ 1 *p* adj < 0.05). This set includes the 93 proteins enriched in ADEVs versus bulk EVs and cell type‐specific EVs, plus the 133 proteins enriched only compared to cell type‐specific EVs. (B) Venn Diagram illustrating the overlap of proteins meeting the stringent functional threshold criteria (FC ≥ 2 *p* adj < 0.05) when comparing ADEVs to bulk EVs and cell type‐specific EVs. The diagram highlights the resultant 66 highly upregulated ADEV proteins. (C) Protein‐protein Interaction (PPI) network showing the interaction map of the 66 highly upregulated ADEV proteins, confirming their unique molecular profile relative to all control groups.

**Data S1:** Supporting Information.

**Table S1:** 118 identified proteins in IgG control.

**Table S2:** Identified proteins across cell type‐specific EVs and bulk EVs.

**Table S3:** Comparison between the common proteins found in NDEVs, MDEVs, ADEVs and bulk EVs, and the proteins found in the IgG control.

**Table S4:** Upregulated proteins in NDEVs compared to bulk EVs and cell type‐specific EVs.

**Table S5:** Upregulated proteins in MDEVs compared to bulk EVs and cell type‐specific EVs.

**Table S6:** Upregulated proteins in ADEVs compared to bulk EVs and cell type‐specific EVs.

## Data Availability

The mass spectrometry proteomics data have been deposited to the ProteomeXchange Consortium via the PRIDE (Perez‐Riverol et al. 2025) partner repository with the dataset identifier PXD071055.
