# Supplementary material for: Cell Type‐Specific Extracellular Vesicles in Mouse Brain: Proteomic Signatures Highlight Astrocytic GlialCAM Network and GPCR Enrichment
Source: Glia. 2026 Aug 21;74(10):e70212. doi: 10.1002/glia.70212 (PMC13496119; doi:10.1002/glia.70212)
Supplement: Supplementary file 1 [file GLIA-74-0-s012.pdf]

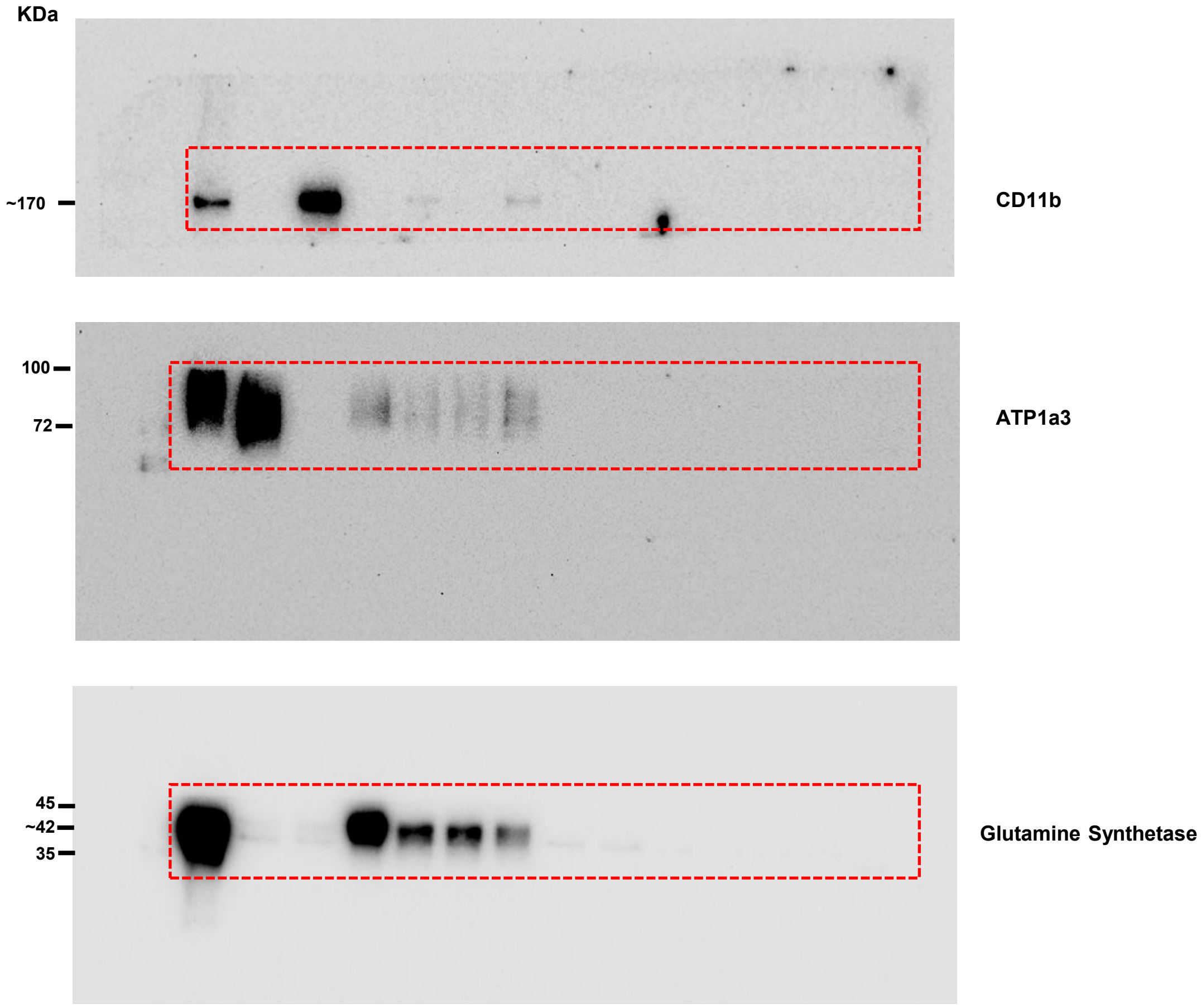

Uncropped Western blot images corresponding to Figure 1A. The same membrane was cut to allow detection of all three markers on a single blot. Red dashed boxes indicate the regions shown in the manuscript figure.

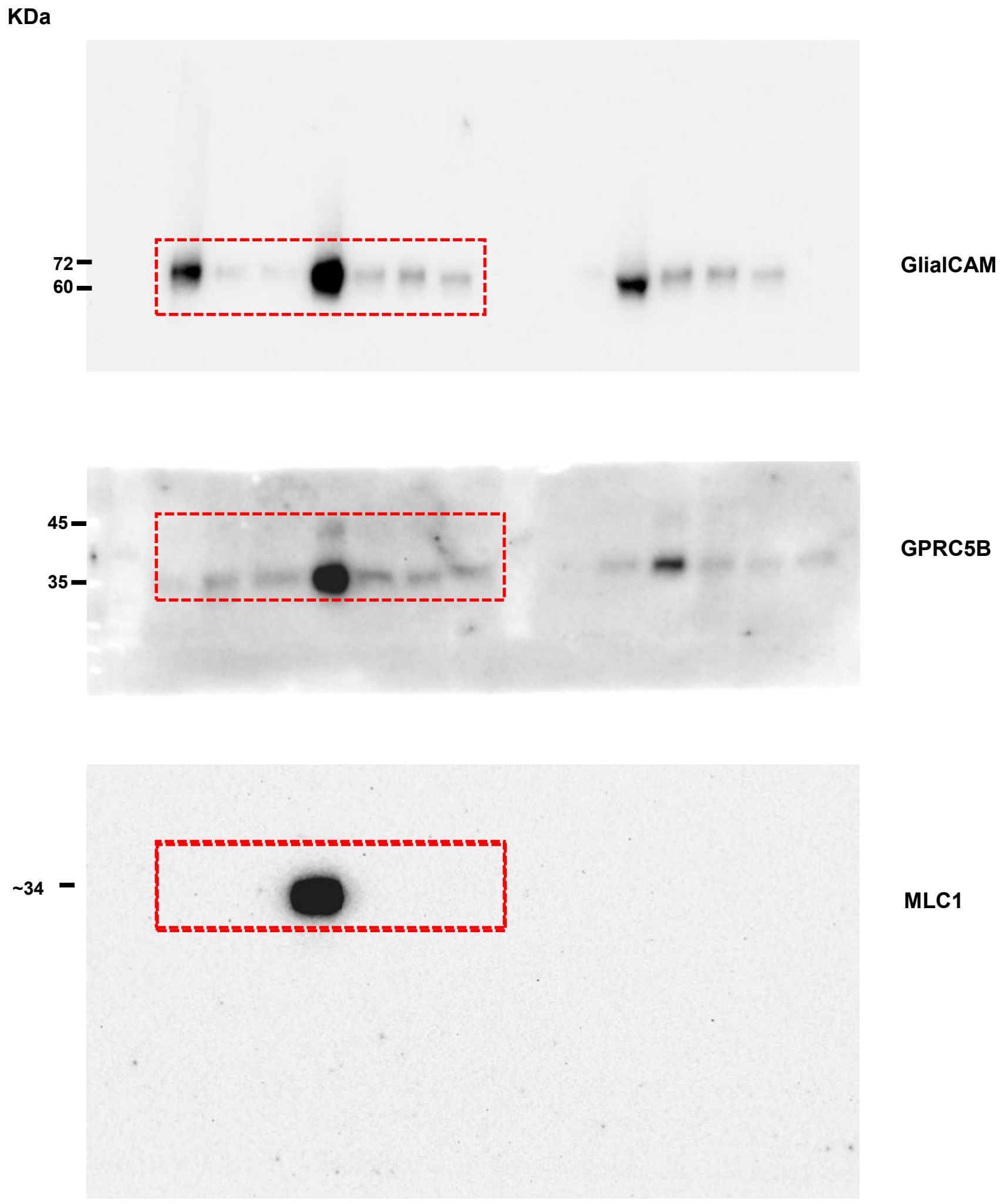

Uncropped Western blot images corresponding to Figure 6B. Membranes were cut to allow detection of additional markers. Red dashed boxes indicate the regions showed in the manuscript figure.

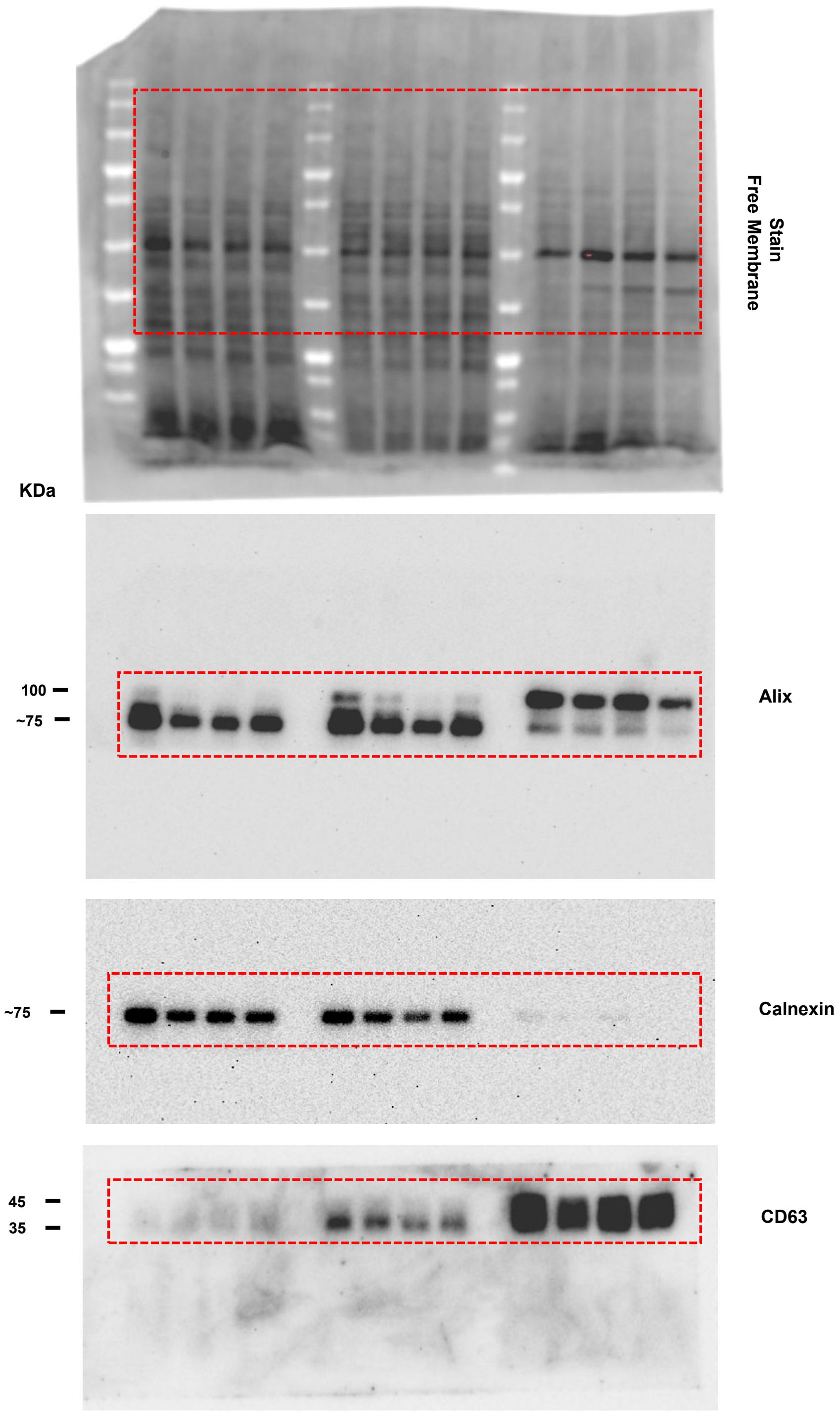

Uncropped Western blot images corresponding to Supplementary Figure 2A. Membranes were cut to allow detection of additional markers. The membrane for Alix was stripped and reprobed to detect calnexin. Red dashed boxes indicate the regions showed in the manuscript figure.

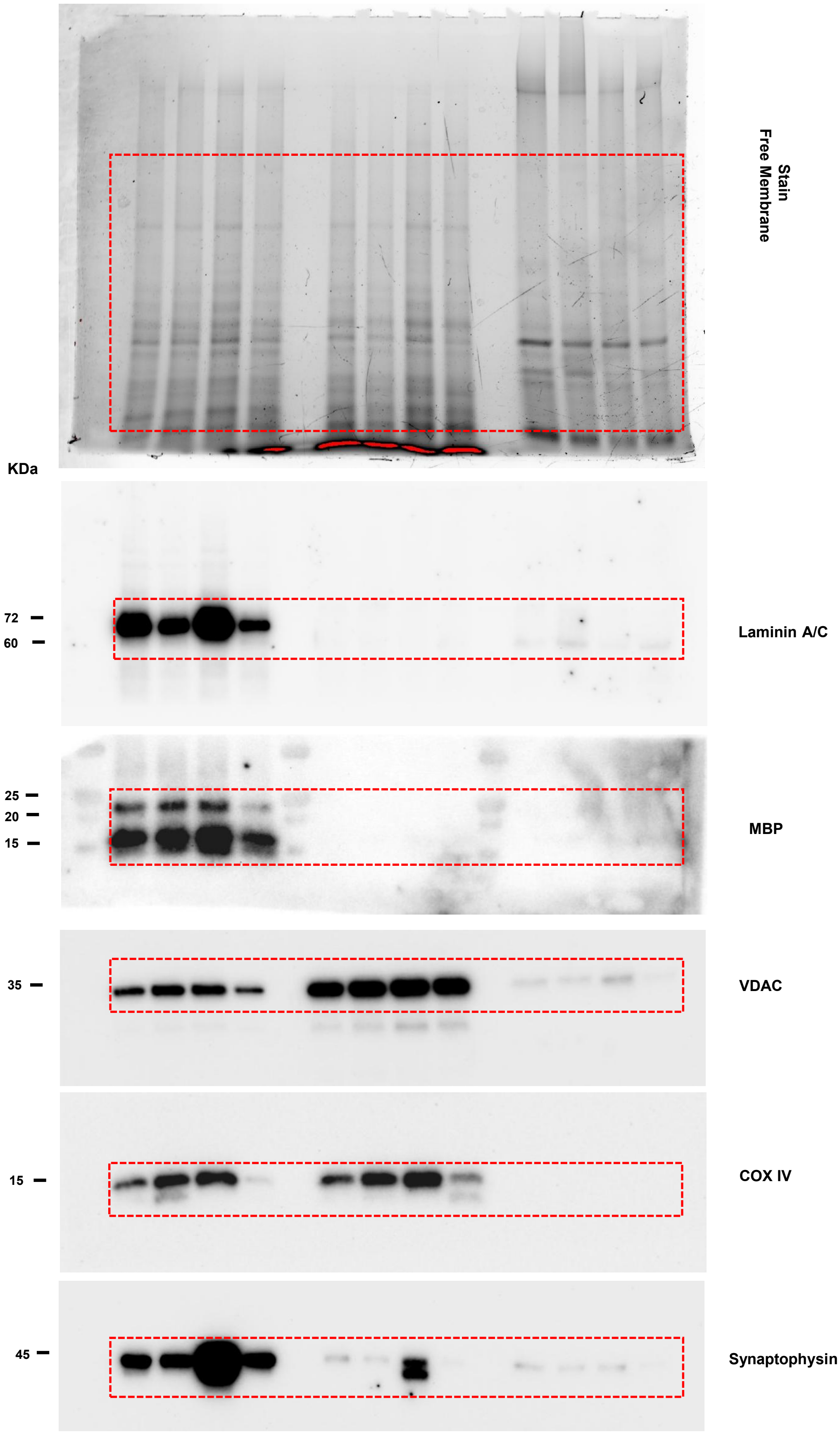

Uncropped Western blot images corresponding to Supplementary Figure 2C. Membranes were cut to allow detection of additional markers. Red dashed boxes indicate the regions showed in the manuscript figure.
